# Mitochondrial and plasma‑membrane transport‑based engineering enables industrial‑level isocitric acid production in *Yarrowia lipolytica*

**DOI:** 10.1186/s12934-026-03098-4

**Published:** 2026-08-26

**Authors:** Eugenia Messina, Cosetta Ciliberti, Evgeniya Yuzbasheva, Serena Barile, Tigran Yuzbashev, Zbigniew Lazar, Ivan Laptev, Ferdinando Palmieri, Luigi Palmieri, Isabella Pisano, Gennaro Agrimi

**Affiliations:** 1 Department of Biosciences, Biotechnology and Environment, University of Bari Aldo Moro, Campus Universitario, via Orabona 4, Bari, 70125 Italy; 2 Both Strands Ltd, St Albans, Hertfordshire AL1 4QW UK; 3 Rothamsted Research, Harpenden, AL5 2JQ UK; 4 Department of Biotechnology and Food Microbiology, Faculty of Biotechnology and Food Science, Wrocław University of Environmental and Life Sciences, Chelmonskiego 37, Wrocław, 51-630 Poland; 5 Alterhof LLC, Melnikova 3, Moscow, 109316 Russian Federation; 6 Interuniversity Consortium for Biotechnology (CIB), Trieste, 34100 Italy

**Keywords:** *Yarrowia lipolytica*, Isocitric acid, Transporters, Fermentation, Mitochondria, Metabolic engineering

## Abstract

**Background:**

Isocitric acid (ICA) has recently attracted increasing interest because of its nutraceutical relevance and emerging biomedical potential. However, despite its value, industrial exploitation of ICA is still limited owing to the lack of cost‑effective and selective microbial production processes. Mitochondrial transporters play a pivotal role in controlling the pool sizes and fluxes of tricarboxylic acid (TCA) cycle intermediates by mediating their selective exchange across the inner mitochondrial membrane. Recently, we identified the mitochondrial carriers *Yl*Yhm2 and *Yl*Sfc1 in *Yarrowia lipolytica* as the specific transporters responsible for citric acid (CA) and ICA efflux, respectively, providing for the first time direct control over the selective secretion of these two organic acids. Building on this discovery, a transporter‑based metabolic engineering strategy was developed to establish a robust and highly selective ICA‑overproducing microbial platform.

**Results:**

Starting from a strain lacking the mitochondrial citrate carrier *YlYHM2* and overexpressing the mitochondrial ICA exporter *YlSFC1*, ICA secretion was further enhanced through overexpression of the plasma‑membrane exporter *YlCEX1*. To further increase mitochondrial carbon availability feeding the TCA cycle, we overexpressed transporters capable of mediating net carbon import into mitochondria, including the dicarboxylate carriers *YlOAC1* and *YlDIC1*, as well as the pyruvate carrier complex subunits *YlMPC1*/*YlMPC2*, either individually or in combination. Among these, *YlOAC1* overexpression resulted in the most pronounced improvement, enabling production of 58.4 ± 2.4 g/L ICA with a molar yield of 0.62 mol/mol glucose in shake‑flask cultures. Fed‑batch bioreactor cultivation of this strain under nitrogen‑limited conditions yielded 152.76 ± 3.2 g/L ICA with a process selectivity of 95% (ICA/CA = 20), representing the highest titers and selectivity reported to date for a ICA producer.

**Conclusions:**

These results clearly demonstrate that precise manipulation of mitochondrial and plasma‑membrane transport processes constitutes a powerful and scalable strategy for redirecting carbon flux toward ICA production. This work establishes *Y. lipolytica* as a robust microbial chassis for industrial ICA manufacturing and highlights selective transport engineering as an effective complementary strategy alongside conventional enzyme centered metabolic engineering approaches, extending and reinforcing previously established roles of mitochondrial carriers in metabolic engineering.

**Supplementary Information:**

The online version contains supplementary material available at https://doi.org/10.1186/s12934-026-03098-4.

## Background

Threo‑DS‑isocitric acid (ICA) is an intermediate of the tricarboxylic acid (TCA) cycle that has recently emerged as a multifunctional bioactive compound with applications spanning medical, nutritional, and industrial fields. In addition to its strong antioxidant and anti‑hypoxic activities, ICA shows potential in the therapeutic management of iron deficiency, enhances cognitive and neuroprotective responses under heavy‑metal exposure, and supports cellular respiration by activating succinate dehydrogenase. Its stress‑protective, hepatoprotective, and metabolic‑regulatory properties further expand its relevance for sports nutrition as well as for the development of new nutraceutical formulations [1]. Despite these promising properties, the broader evaluation and application of ICA remain limited by the challenges associated with its production and the resulting high cost. The naturally occurring form of ICA extracted from *Sedum spectabile*, a species of flowering plant, is extremely expensive (1 g = 780 €; Sigma-Aldrich) and therefore poorly accessible for large-scale studies, including clinical validation of its biological effects. Chemically synthesized ICA, produces a mixture of four stereoisomers that are difficult to separate [2]. This has increased interest in microbial production strategies, which predominantly generate the natural threo‑DS isomer of ICA and can substantially reduce overall production costs.

The non-conventional yeast *Yarrowia lipolytica* has gained attention for its capability to produce high levels of citric acid (CA) and ICA, whose synthesis occurs in mitochondria through TCA cycle. The proportions of these acids depend largely on the strain, carbon source, and growth medium composition [3]. In the past decades, researchers have been actively developing both mutant and genetically engineered yeast strains capable of significantly overproducing ICA. For example, targeted gene modification through aconitase overexpression enabled the construction of a recombinant strain that produced 72.6 g/L of ICA with a process selectivity of 72% in rapeseed oil medium [4]. In parallel, several hyper‑producing strains have been generated through random chemical mutagenesis. Among these, one of the highest‑performing mutants accumulated 88.7 g/L of ICA with a process selectivity of 86%, when cultivated in rapeseed oil medium [5]. However, despite the high titres that may occasionally be achieved through random approaches, the lack of a clear understanding of the underlying mutational background and associated mechanisms makes rational metabolic engineering the most reliable and controllable strategy for constructing stable, high‑performing ICA‑producing strains.

Among the various metabolic engineering strategies explored to enhance ICA production, increasing attention has been directed toward transporters involved in metabolite exchange between cellular compartments. Since ICA biosynthesis takes place in the mitochondria, its production critically depends on the efficient shuttling of metabolic intermediates between the mitochondrial matrix and the cytosol. Mitochondrial transporters constitute key regulatory nodes in eukaryotic metabolism, functioning as critical control points for substrate supply and product export, and their overexpression or deletion can markedly reshape metabolic flux distributions [6]. Consequently, mitochondrial carriers have emerged as strategic targets for optimizing pathway efficiency, boosting metabolic fluxes, and enhancing biotechnological ICA yield and titer.

The inner mitochondrial membrane is impermeable to both ICA and CA. Two mitochondrial carriers involved in the specific transport of CA and ICA to the cytosol, were recently identified: *Yl*Yhm2 for CA export and *Yl*Sfc1 for ICA efflux across the mitochondrial membrane. Overexpressing *YlYHM2* gene together with adenosine monophosphate deaminase (*YlAMPD*) gene markedly enhanced CA production, yielding 97.1 g/L with 94% selectivity [7]. Conversely, the overexpression of *YlSFC1* shifted the ICA/CA ratio toward ICA. Furthermore, combining *YlSFC1* and *YlAMPD* overexpression with *YlYHM2* inactivation, enabled the production of 136.7 g/L of ICA with 88% selectivity in a glucose fed‑batch cultivation [8] .

The aim of this work was to construct an even more efficient recombinant strain *of Y. lipolytica* capable of producing ICA as its main product. Building on previous findings regarding mitochondrial carriers involved in CA and ICA transport, we used as a starting point a strain in which the *YlYHM2* gene was inactivated, and *YlSFC1* gene was overexpressed. To further enhance ICA production, our first engineering strategy focused on improving metabolite export across the plasma membrane. For this purpose, we overexpressed *YlCEX1* gene encoding for a transporter previously identified as the major CA exporter [9]. The second metabolic engineering strategy focused on mitochondrial transporters that channel carbon into the TCA cycle. Building on previously reported strategies targeting mitochondrial transporters, redirecting carbon flux toward mitochondria represents a promising approach for enhancing microbial organic acids production: by increasing the availability of early TCA intermediates through anaplerotic reactions, mitochondrial carbon flow is strengthened, ultimately supporting higher titres of TCA-derived products [10]. Three mitochondrial transporters, responsible for delivering carbon to mitochondria, were overexpressed individually or in combination: the oxaloacetate carrier, the dicarboxylate carrier, and the pyruvate carrier. Among the transporters tested, overexpression of the gene encoding for the mitochondrial oxaloacetate carrier *Yl*Oac1, in combination with *YlYHM2* gene deletion and overexpression of genes *YlSFC1* and *YlCEX1*, yielded the highest ICA-producing microbial strain reported to date. This strain was able to produce up to 152.76 ± 3.2 g/L ICA with 95% process selectivity (ICA/CA = 20) in a fed-batch bioreactor using a pulse-feeding strategy, showing its suitability for industrial ICA production.

## Results

### *YlCEX1* overexpression boosts isocitric acid production

**Fig. 1:**
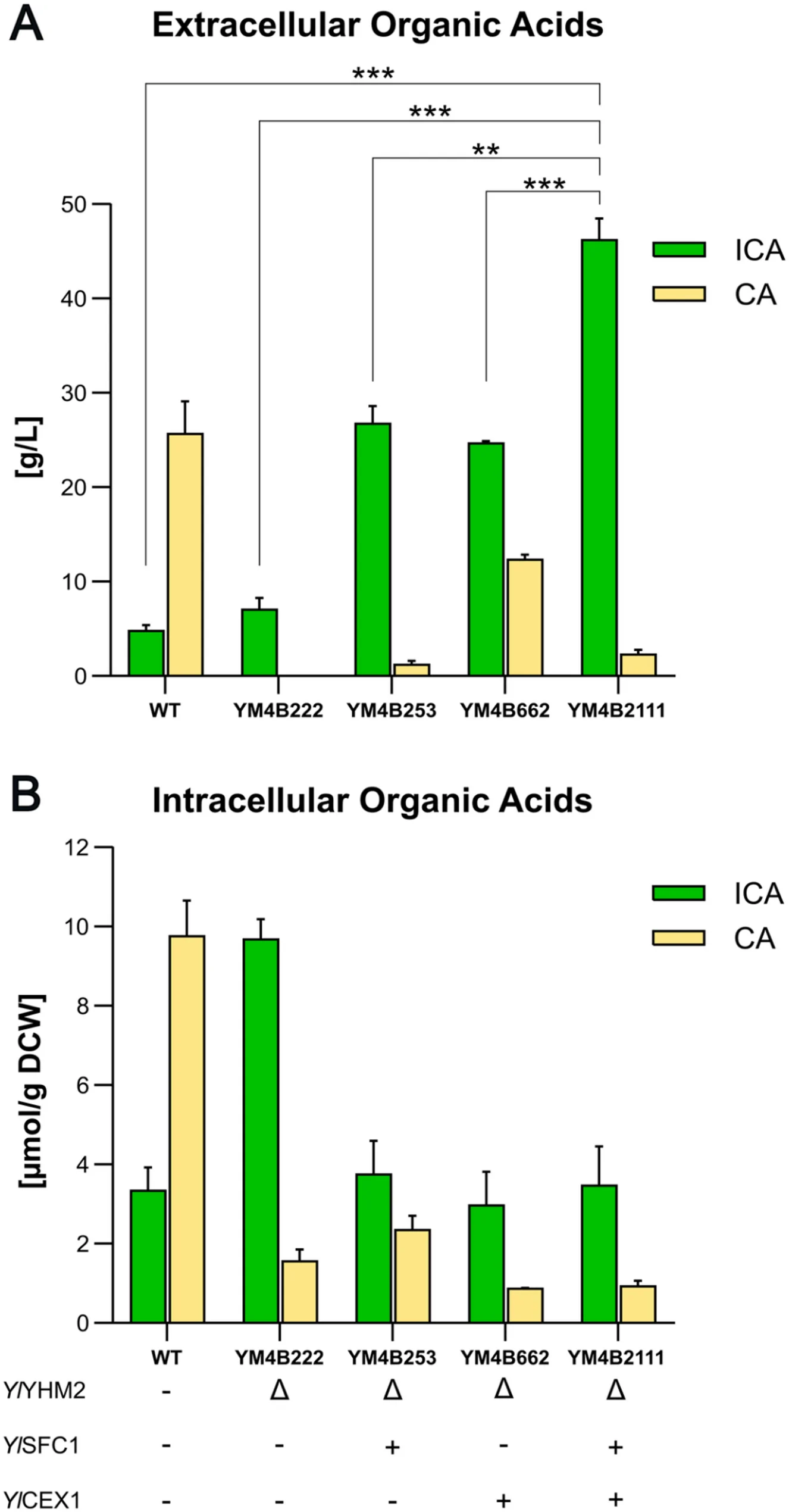
Extracellular and intracellular accumulation of isocitric acid (ICA, green) and citric acid (CA, yellow) in the indicated *Y. lipolytica* strains after 4 days of cultivation in 250-mL baffled flasks containing 50 mL YNB medium supplemented with 90 g/L glucose and 2 g/L (NH₄)₂SO₄. (**A**) Extracellular organic acid concentrations (g/L). (**B**) Intracellular organic acid levels (µmol/g DCW). Data represent the mean of at least three independent experiments, and error bars indicate the standard error. Statistical significance was determined using an unpaired Student’s *t*-test (**P < 0.01; ***P < 0.001)

During growth on glucose, ICA biosynthesis primarily occurs within the mitochondrial matrix in *Y. lipolytica*. Consequently, efficient secretion requires transport of ICA across both the inner mitochondrial membrane and the plasma membrane to reach the extracellular environment. As a starting point, we selected *Y. lipolytica* wild-type strain Y-4973 (WT; Table 3), carrying the Δ*ura3* and Δ*ku70* deletions and suitable for targeted integrations at defined chromosomal loci using the CRISPR/Cas9‑based DNA assembly toolkit *YaliCraft*^®^ [11]. Based on our previous findings identifying key transport determinants of ICA production [8], these transport‑related genetic modifications were implemented anew in the Y‑4973 genetic background. In particular, inactivation of the mitochondrial citrate carrier gene *YlYHM2* was introduced, resulting in strain YM4B222 (Table 3) and leading to a complete abolition of CA secretion (Fig. 1A). The disruption of the *YlYHM2* gene had been expected to promote the accumulation of CA inside mitochondria, where it could be converted into ICA and subsequently exported to the cytosol. It is noteworthy that this initial genetic modification did not lead to an increase in ICA production, compared to WT strain (Fig. 1A), despite the fact that CA could no longer be transported out of the mitochondria. This may imply that ICA secretion is primarily limited by specific transporter‑dependent mechanisms.

The second modification involved the overexpression of the mitochondrial carrier gene *YlSFC1*, and resulted in strain YM4B253 (∆*Ylyhm2 YlSFC1*) (Table 3). The combination of these two modifications increased ICA production in flask cultivation on glucose to 26.8 ± 1.8 g/L, corresponding to approximately 6.0-fold and 4.0-fold increases relative to the WT and YM4B222 strains, respectively (Fig. 1A). These results are consistent with our previous findings [8].

ICA production is likely limited by the efficiency of its export across the plasma membrane. In particular, the identification of *Yl*Cex1 as a common transporter for both CA and ICA, along with evidence that its overexpression stimulates the synthesis of both metabolites [9].

Notably, overexpression of *YlCEX1* in the YM4B222 (*Δyhm2*) strain restored CA synthesis in the resulting YM4B662 strain (Fig. 1A) and led to a 3.5-fold increase in secreted ICA levels (24.4 ± 0.2 g/L) compared to the parental strain YM4B222 (Table 1A). These results demonstrate the specific role of *Yl*Cex1 in the transport of both organic acids, CA and ICA.

These additional results further supported the rationale for *YlCEX1* overexpression in YM4B253, leading to the construction of strain YM4B2111 (*Δyhm2 YlSFC1 YlCEX1*) (Table 3). In this strain, the ICA titer obtained in flask cultures significantly increased by approximately 10-, 6.5- and 1.7-fold compared to the WT, YM4B222 and YM4B253 strains, respectively, achieving a final titer of 46.2 ± 2.3 g/L (Fig. 1A). The overexpression of *YlCEX1* gene combined with inactivation of *YlYHM2* and overexpression of *YlSFC1* resulted in a 66‑fold increase in the ICA/CA ratio compared to the WT strain (Table 1). In addition, this engineering strategy yielded the highest molar yield from glucose (0.54 ± 0.04 mol/mol), reaching a value approximately 9.0- and 1.6-fold higher than that of the WT and YM4B253, respectively (Table 1).

**Table 1:** Biomass (g/L DCW), citrate and isocitrate (g/L), ICA/CA ratio and molar yield (Y_ICA/GLU_) after 4 days of flask cultivation

Strain^1^	Biomass (g/L DCW)	Citrate (g/L)	Isocitrate (g/L)	Ratio ICA/CA	Y_ICA/GLU_ (mol/mol)
WT	11.7 ± 1.2	22.9 ± 1.6	4.8 ± 0.6	0.2	0.05 ± 0.01
YM4B222	15.5 ± 1.4	N.D.	7.1 ± 0.5	N.D.	0.08 ± 0.01
YM4B253	9.4 ± 1.1	1.2 ± 0.4	26.8 ± 1.8	21.7	0.33 ± 0.07
YM4B662	12.6 ± 0.6	12.4 ± 0.5	24.7 ± 0.2	2	0.29 ± 0.00
YM4B2111	12.1 ± 1.0	2.3 ± 0.4	46.2 ± 2.3	19.8	0.54 ± 0.04

^1^ WT (W29 Δ*ku*70 Δ*ura3*); YM4B222 (W29 Δ*ku*70 ∆*Ylyhm2* Δ*ura3*); YM4B253 (W29 Δ*ku*70 ∆*Ylyhm2 YlSFC1*); YM4B662 (W29 Δ*ku*70 ∆*Ylyhm2 YlCEX1*); YM4B2111 (W29 Δ*ku*70 ∆*Ylyhm2 YlSFC1 YlCEX1*). Data represent the mean of at least three independent experiments, with error bars indicating the standard error

Overall, the concomitant overexpression of the transporters primarily involved in ICA efflux, the mitochondrial carrier *Yl*Sfc1 and the plasma membrane transporter *Yl*Cex1, acted synergistically to significantly increase ICA production, while deletion of the mitochondrial citrate carrier *YlYHM2* was employed as an additional strategy to further enhance ICA export.

To determine whether engineering carboxylate transporters also altered intracellular organic-acid pools, we quantified intracellular CA and ICA levels in all engineered strains (Fig. 1B). In the wild-type, the intracellular pool was largely dominated by CA (9.8 ± 0.9 µmol/g DCW), whereas ICA accumulated at lower levels (3.3 ± 0.6 µmol/g DCW), resulting in an ICA/CA ratio of 0.34. Deletion of *YlYHM2*, completely reversed this balance in all engineered strains, with ICA/CA ratios consistently above 1. The strongest effect was observed in YM4B222 (Δ*Ylyhm2*), which accumulated the highest intracellular ICA concentration among all strains (9.7 ± 0.5 µmol/g DCW) while maintaining a markedly reduced CA pool (1.6 ± 0.3 µmol/g DCW) (Fig. 1B). As a result, the ICA/CA ratio increased to 6.2. Overexpression of the mitochondrial ICA carrier *YlSFC1* (YM4B253), the plasma-membrane tricarboxylate exporter *YlCEX1* (YM4B662), or both genes simultaneously (YM4B2111) reduced intracellular ICA levels relative to YM4B222, yielding values between 3.0 and 3.8 µmol/g DCW (Fig. 1B). CA concentrations remained low (0.9–2.4 µmol/g DCW), preserving the inverted ICA/CA balance, although to a lesser extent (ratios of 1.6–3.7).

Notably, intracellular CA levels were significantly higher in the wild-type than in any strain carrying the *YlYHM2* deletion, and this phenotype was not substantially affected by overexpression of either *YlSFC1* or *YlCEX1*.

### Enhancing isocitric acid production by engineering mitochondrial carbon-import transporters

**Fig. 2:**
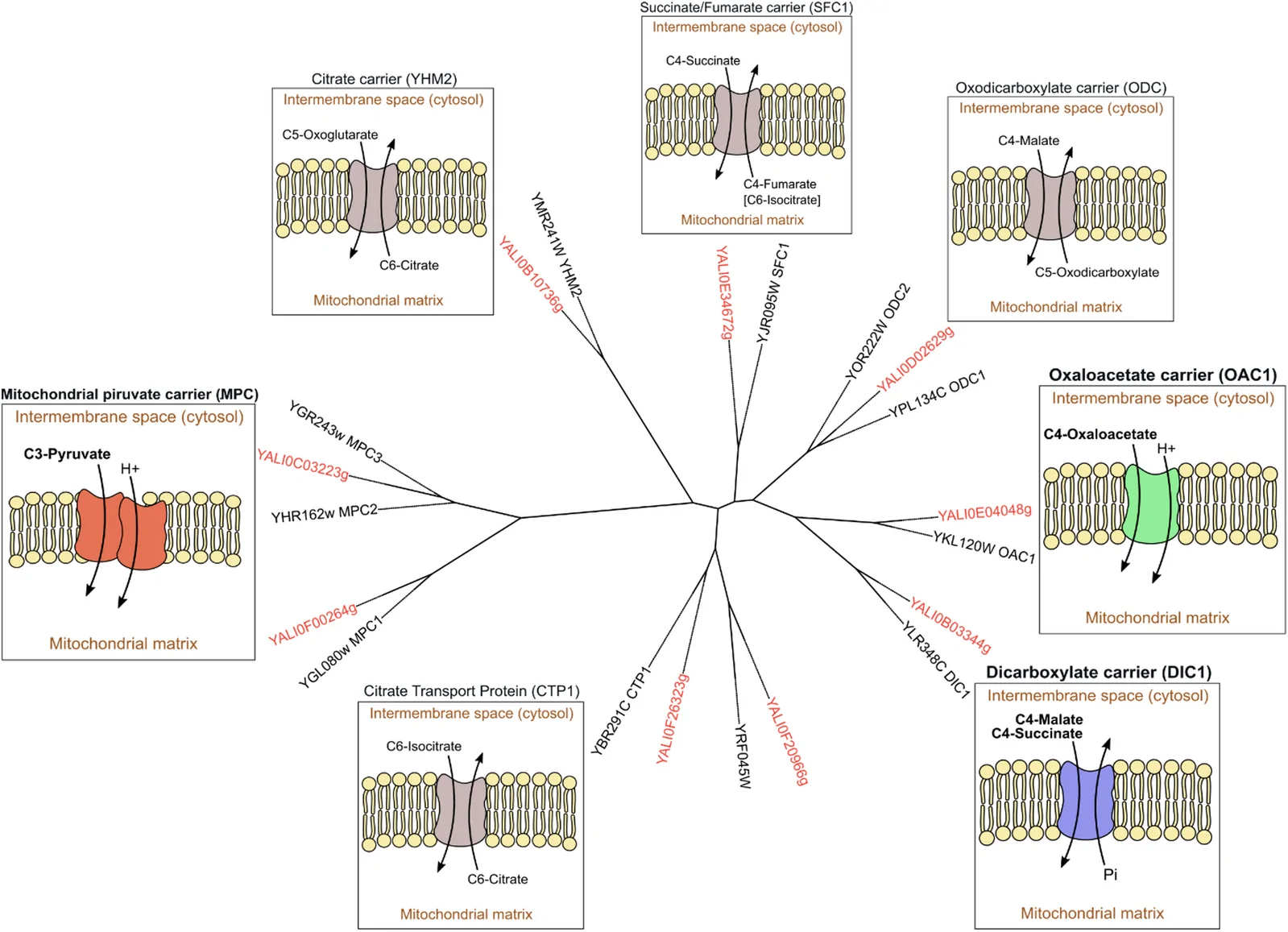
Phylogenetic tree of mitochondrial carriers of *S. cerevisiae* and *Y. lipolytica*. The tree was constructed using the sequences of di- ad tri-carboxylate mitochondrial transporters and MPC complex subunits from *S. cerevisiae* (black) and *Y. lipolytica* (red). The names of the mitochondrial transporters and/or their coding genes are found on the terminal nodes

Mitochondrial organic acids transport is mediated by several carriers, including the tricarboxylate, dicarboxylate, succinate/fumarate, oxoglutarate, and oxo dicarboxylate transporters. These systems function almost exclusively as strict carboxylate exchangers, translocating carbon skeletons across the inner mitochondrial membrane without generating a net influx of carbon into the matrix; some of these transporters can even mediate a net efflux of carbon from the mitochondria. Only a minority of mitochondrial carriers can mediate a net carbon import [12] (Fig. 2). This feature is particularly relevant for anaplerosis, the process by which cells replenish TCA cycle intermediates when these are diverted to biosynthetic pathways. In this context, we evaluated whether boosting mitochondrial carbon replenishment, through the overexpression of gene encoding for transporters mediating net carbon import, could increase the availability of TCA intermediates required for ICA synthesis.

These carriers have not yet been characterized in *Y. lipolytica*, but their function is known in *S. cerevisiae*. The oxaloacetate carrier (*Sc*Oac1) transports cytosolic oxaloacetate (OAA) through two mechanisms, strict substrate exchange of di- and/or tri-carboxylates and H⁺-coupled symport, collectively enabling its net mitochondrial uptake [13] (Fig. 2). The mitochondrial dicarboxylate carrier (*Sc*Dic1) catalyses a strict counter-exchange between dicarboxylates (malate, succinate, fumarate) and inorganic phosphate or sulphate [14] (Fig. 2). The mitochondrial pyruvate carrier (Mpc) is a multi-subunit complex composed of *Sc*Mpc1, *Sc*Mpc2, and *Sc*Mpc3, which together are essential for importing pyruvate from the cytosol into mitochondria through a proton-coupled symport mechanism [15] (Fig. 2). Their expression is regulated in a carbon‑source-dependent manner. Under fermentative conditions, *Sc*Mpc1 associates with *Sc*Mpc2 to form the MPC_FERM_ complex, whereas under respiratory conditions, *Sc*Mpc1 interacts with *Sc*Mpc3 to assemble the MPC_OX_ complex [16, 17]. In contrast to Oac1 and Dic1, which are classified within the conserved canonical solute carrier family 25A (SLC25), the Mpc proteins are distinct and do not belong to the SLC25 family [18].

*Y. lipolytica* protein database was screened for sequences of di- and tri-carboxylate mitochondrial transporters and Mpc complex, obtaining 9 sequences. A phylogenetic tree (Fig. 2) was built using these sequences along with the corresponding *S. cerevisiae* homologs, for all of which a function has been assigned except for YRF045W. As a result, at least one *Y. lipolytica* putative ortholog has been assigned for each transport function.

In *Y. lipolytica*, Oac1 is encoded by YALI0E04048g (RefSeq XP_503525), whereas Dic1 is encoded by YALI0B03344g (RefSeq CAG82683.1) [19, 20] (Fig. 2). In this work, these genes were named *YlOAC1* and *YlDIC1*. Regarding the pyruvate carrier, a BLASTp search using the three *S. cerevisiae* subunits as queries identified only two proteins in *Y. lipolytica*, RefSeq XP_504811.1 and XP_501390.1, corresponding to YALI0F00264g and YALI0C03223g encoding genes, respectively (Fig. 2). YALI0F00264p shares the highest amino acid identity (63.7%) with *Sc*Mpc1, and the corresponding gene was therefore designated as *YlMPC1*. YALI0C03223p shows the highest amino acid identity (59.0%) with *Sc*Mpc3, but its gene was named *YlMPC2* with which it shares the 56.6% amino acid identity (Table S1, Supplementary Materials).

**Fig. 3:**
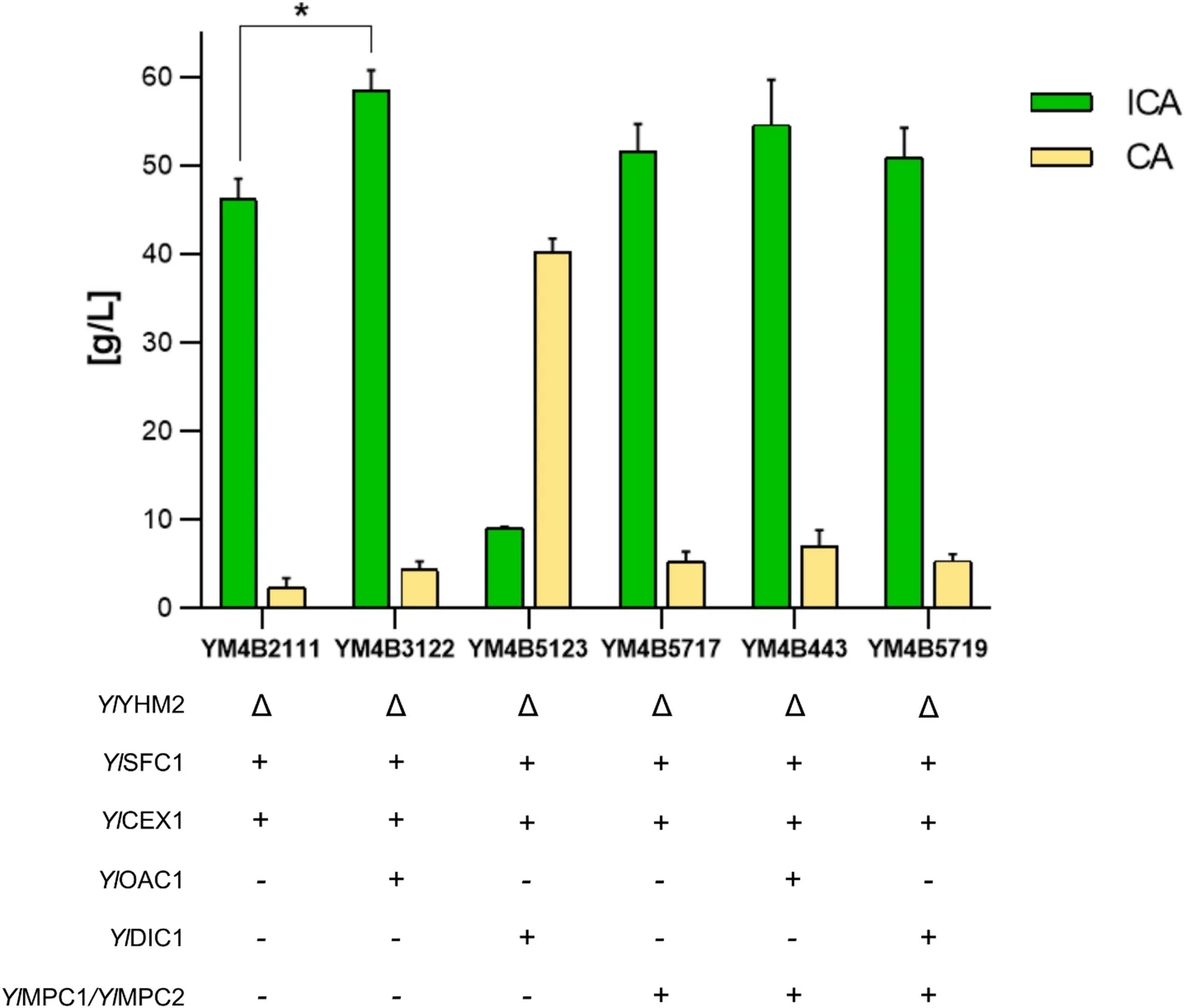
Isocitric acid (green) and citric acid (yellow) production after 4 days of cultivation in 250-mL baffled flasks with 50 mL YNB+Glucose 90 g/L and nitrogen starvation [(NH_4_)_2_SO_4_ 2 g/L]. Data represent the mean of at least two independent experiments, with error bars indicating the standard error. The statistical significance was determined using an unpaired Student’s t-test (*P < 0.05)

The genes encoding the three mitochondrial transporters *YlOAC1*, *YlDIC1* and *YlMPC1*/*YlMPC2* were overexpressed individually or in combination in the strain YM4B2111 (W29 Δ*ku*70 ∆*Ylyhm2 YlSFC1 YlCEX1*). Individual overexpression of *YlOAC1*, *YlDIC1* and *YlMPC1*/*YlMPC2* generated the strains YM4B3122, YM4B5123 and YM4B5717, respectively (Table 3). The same genes were then co‑overexpressed to assess potential synergistic effects, yielding the strains YM4B443 (*YlMPC1/YlMPC2* + *YlOAC1*) and YM4B5719 (*YlMPC1*/*YlMPC2* + *YlDIC1*) (Table 3). All combinations resulted in increased ICA production compared to the YM4B2111 parental strain, except for the single *YlDIC1* overexpression (YM4B5123), which caused an inversion of the ICA/CA ratio in favour of CA (Fig. 3; Table 2). Interestingly, this effect was not observed when *YlDIC1* gene overexpression was combined with that of *YlMPC1*/*YlMPC2* genes in strain YM4B5719 (Fig. 3). Among all the tested genetic combinations, strain YM4B3122, overexpressing *YlOAC1*, showed the most pronounced improvement. In this strain, the ICA titer increased by approximately 1.3‑fold, reaching 58.4 ± 2.4 g/L, compared with the parent strain YM3B2111. The statistical significance (*P* = 0.03) of this difference was confirmed in unpaired Student’s t-test (Fig. 3). Moreover, YM4B3122 exhibited the highest molar yield on glucose among all recombinant strains, achieving 0.62 ± 0.01 mol ICA/mol glucose (Table 2). No significant differences were detected in biomass accumulation (Table 2). Moreover, in the YM4B443 strain, the simultaneous overexpression of *YlMPC1*/*YlMPC2* and *YlOAC1* genes resulted in the second-highest ICA production and molar yield from glucose, among all the strains tested (Table 2). Since the ICA/CA ratio worsens in this strain (Table 2), we overexpressed an additional gene copy of *YlSFC1*, generating strain YM4B464 (Table 3), in an effort to further enhance ICA secretion. The ICA titer decreased by approximately 1.4‑fold compared to the parental strain (Figure S1, Supplementary Materials), indicating that this additional genetic modification was not beneficial for ICA production.

Notably, manipulation of mitochondrial carriers presumed to enhance mitochondrial carbon supply, increased ICA production across all strains, while concomitantly elevating CA synthesis by up to 3.0-fold (Fig. 3; Table 2), consistent with a more efficient carbon supply. Although in all strains the combined amount of ICA and CA was higher than in the parental strain YM4B2111, only the difference between this strain and YM4B3122 was statistically significant (*P* = 0.01).

**Table 2:** Biomass (g/L DCW), citrate and isocitrate (g/L), ICA/CA ratio and molar yield (Y_ICA/GLU_) after 4 days of flask cultivation

Strain^1^	Biomass (g/L DCW)	Citrate (g/L)	Isocitrate (g/L)	Ratio ICA/CA	Y_ICA/GLU_ (mol/mol)
YM4B2111	12.6 ± 1.4	2.3 ± 0.4	46.2 ± 2.3	19.8	0.54 ± 0.04
YM4B3122	14.0 ± 1.1	4.4 ± 0.9	58.4 ± 2.4*	13.4	0.62 ± 0.01
YM4B5123	13.0 ± 0.6	40.3 ± 1.4	9.0 ± 0.2	0.2	0.10 ± 0.00
YM4B5717	14.6 ± 0.3	5.2 ± 1.2	51.6 ± 3.1	9.9	0.57 ± 0.02
YM4B443	14.0 ± 1.0	6.9 ± 1.8	54.6 ± 5.1	7.9	0.59 ± 0.03
YM4B5719	13.3 ± 1.1	5.3 ± 0.8	50.9 ± 3.3	9.6	0.57 ± 0.02

^1^YM4B2111 (W29 Δ*ku70* ∆*Ylyhm2YlSFC1YlCEX1*); YM4B3122 (W29 Δ*ku70 *∆*Ylyhm2YlSFC1YlCEX1YlOAC1*); YM4B5123 (W29 Δ*ku70* ∆*Ylyhm2YlSFC1 YlCEX1 YlDIC1*); YM4B5717 (W29 Δ*ku70* ∆*Ylyhm2YlSFC1YlCEX1YlMPC1*/*YlMPC2*); YM4B443 (W29 Δ*ku70* ∆*Ylyhm2YlSFC1 YlCEX1 YlOAC1YlMPC1*/*YlMPC2*); YM4B5719 (W29 Δ*ku70* ∆*Ylyhm2 YlSFC1 YlCEX1 YlDIC1YlMPC1*/*YlMPC2*. Data represent the mean of at least two independent experiments, with error bars indicating the standard error. The statistical significance was determined using an unpaired Student’s t-test (*P < 0.05)

In *Y. lipolytica*, the production of CA and ICA is enhanced under nitrogen‑limiting conditions in the presence of excess carbon. One of the mechanisms involved in accumulation of organic acids in a nitrogen starvation environment, involves the activity of adenosine monophosphate deaminase (*YlAMPD*) that release ammonium from adenosine monophosphate (AMP) to compensate for nitrogen depletion. Since AMP is an allosteric activator of isocitrate dehydrogenase (*IDH*), the function of the *YlAMPD* results in a partially interrupted TCA cycle, determining CA and ICA accumulation in the mitochondria [8]. We therefore overexpressed the *YlAMPD* gene in the best isocitrate hyper-producing strain, YM4B3122 (W29 Δ*ku*70 ∆*Ylyhm2 YlSFC1 YlCEX1 YlOAC1*), to assess whether this additional genetic modification could further increase the amount of secreted ICA. The resulting strain YM4B481 (Table 3) however, showed a 15% decrease in ICA production compared with the parental strain (49.3 ± 2.6 g/L) (Figure S2, Supplementary Materials).

Based on its highest ICA titer and molar yield, strain YM4B3122 was selected for bioreactor‑scale ICA production. This choice was further supported by the observation that single overexpression of *YlOAC1* resulted in the highest ICA/CA ratio among the newly tested strains (Table 2).

### Improved isocitrate production of YM4B3122 via optimized fed-batch cultivation

For bioreactor cultivation, a fed-batch process operated with a pulse-feeding strategy was adopted, as this approach has previously been demonstrated to be an effective strategy to enhance ICA production during scale-up in *Y. lipolytica* strains [5, 4, 21].

**Fig. 4:**
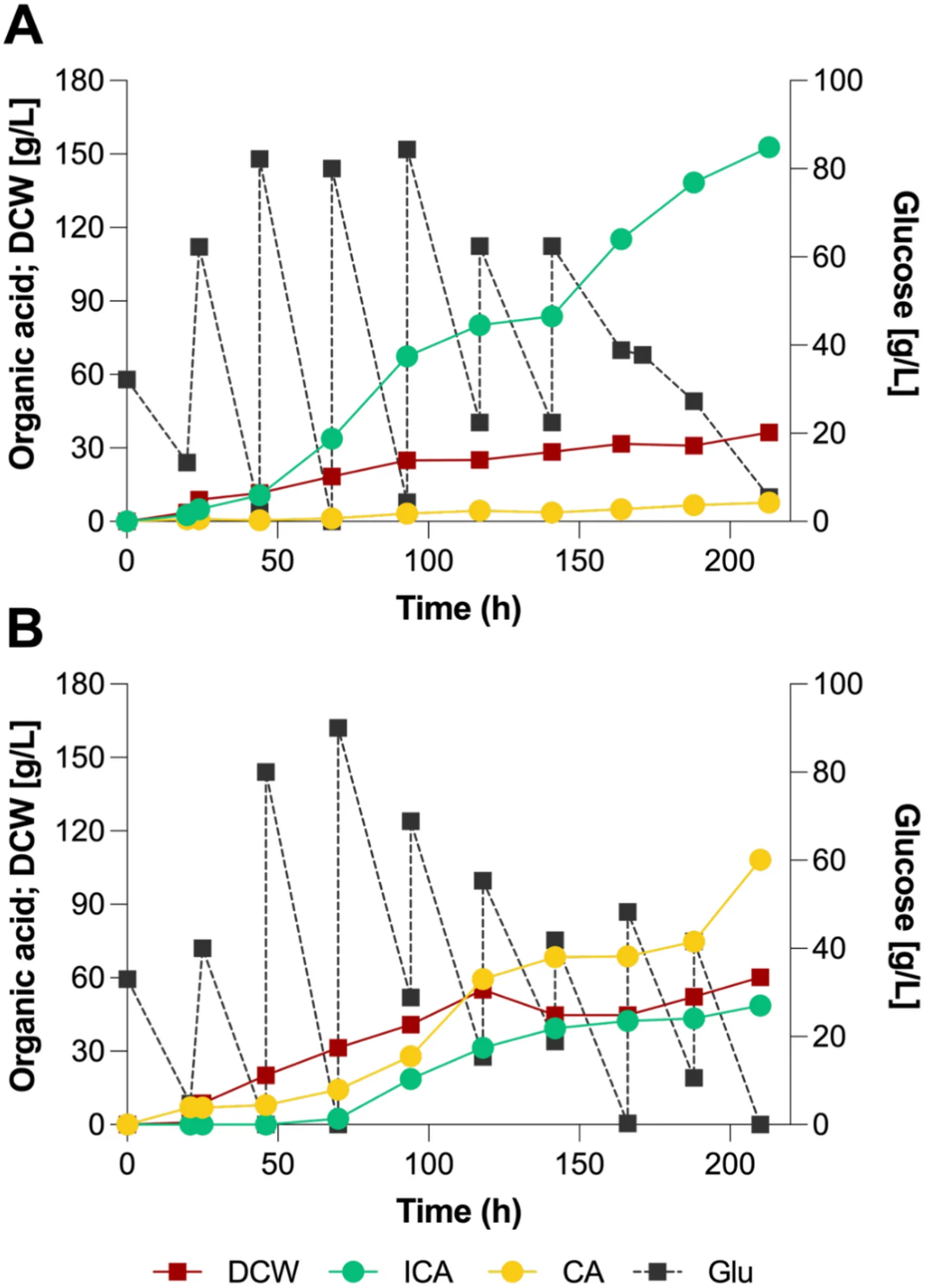
Fed-batch bioreactors cultivations of YM4B3122 ICA-overproducing strain (**A**) and of WT strain Y-4973 (**B**). These cultivations were carried at 28 °C with agitation rate of 800 rpm, air flow of 0.8 vvm and pH set at 6, inoculated with an initial OD_600nm_ of 1.0. These cultivations were carried out in two replicates (Figure S3, Supplementary Materials) and the data depict a single representative cultivation

The bioreactor cultivation of strain YM4B3122 started with a 24 h batch phase carried out with 30 g/L glucose and 3 g/L (NH_4_)_2_SO_4_ (C/N 19), a condition selected to ensure almost complete substrate consumption and biomass formation. After this initial batch phase, a pulse-feeding strategy was employed for the fed-batch cultivation. Glucose feeding was adjusted daily based on the estimated 24 h consumption with a 10% increment, introduced to account for the progressively increasing glucose demand associated with biomass accumulation and maintenance in the bioreactor. (NH_4_)_2_SO_4_ was co-fed to maintain a substrate C/N ratio of 94, ensuring nitrogen-limited conditions required for ICA accumulation, as previously demonstrated [8]. In addition, nitrogen supplementation ensured efficient glucose consumption also during the late stages of cultivation, allowing the process to be prolonged up to 213 h. Preliminary experiments (data not shown) indicated that pulse feeding of glucose alone, resulted in inefficient substrate utilization after several pulse-feeding, ultimately forcing premature termination of the cultivation. During the cultivation, glucose was consumed at an average rate of 2.96 g/L/h between 0 and 117 h, with the highest consumption rate (3.43 g/L/h) observed between 44 and 68 h. ICA productivity reached a peak of 1.53 g/L/h between 141 and 168 h of cultivation with an average productivity of 0.72 g/L/h throughout the entire 213 h-cultivation. Under these conditions, strain YM4B3122 achieved an ICA titer of 152.76 g/L at the end of the cultivation (Fig. 4A), with a final ICA yield (Y_P/S_) of 0.34 mol/mol. ICA selectivity over CA reached 95%, corresponding to an ICA/CA ratio of 20. Accordingly, CA was produced only at low levels (7.65 g/L), with a CA yield (Y_P/S_) of 0.02 mol/mol from glucose.

The same cultivation strategy was applied to the WT strain as a control. Under identical process conditions, the WT strain accumulated predominantly CA, displaying an ICA/CA ratio of 0.5. Final titres of 108.3 g/L CA and 48.6 g/L ICA were obtained (Fig. 4B), corresponding to product yields (Y_P/S_ ) of 0.24 and 0.11 mol/mol glucose, respectively.

## Discussion

ICA is a valuable compound with potential applications in the pharmaceutical and nutraceutical sectors; however, its broader utilization is hindered by the high cost of its manufacture. Due to limitations in its production by chemical synthesis as well as extraction from plant sources, a biotechnological route represents a promising alternative. In this study, previously validated microbial platform based on *Y. lipolytica* [8], was optimized achieving the highest ICA production reported to date. The strategy was primarily based on the engineering of metabolite transport and began with the manipulation of two mitochondrial carriers, *Yl*Yhm2 and *Yl*Sfc1, which have been shown to play a key role in modifying the ICA/CA ratio in *Y. lipolytica* [7, 8]. In this work, the inactivation of *YlYHM2* gene combined with overexpression of the *YlSFC1* gene, enhanced ICA production in the resulting strain YM4B253, with an increase of 5.6-fold compared to the WT strain (Fig. 1A; Table 1). The ICA/CA ratio was shifted towards ICA production, confirming the key role of *Yl*Sfc1 in ICA transport across the mitochondrial membrane. In our previous work, gene overexpression was achieved through random multicopy integration mediated by the non‑homologous end‑joining (NHEJ) repair mechanism [8]. However, in many non‑conventional yeasts, where NHEJ represents the predominant DNA repair pathway, this approach can result in less precise genomic insertions and may lead to reduced genetic stability [22, 23]. In the present work, the same genetic modifications were achieved using the synthetic biology toolkit *YaliCraft*^®^, based on CRISPR/Cas9 technology [11], enabling targeted overexpression at defined chromosomal loci. Locus-specific integration avoids the strong position effects commonly associated with random insertions in *Y. lipolytica*, which often lead to unpredictable and highly variable transcriptional outputs. As reported, randomly integrated constructs frequently undergo phenotypic drift, whereas strains generated through targeted homologous recombination display significantly greater genetic and phenotypic stability [24]. In addition, random integration can result in the formation of unstable tandem repeats that are prone to recombination and gene loss under metabolic stress. These limitations further emphasize the robustness of locus-specific integration strategies for ensuring stable gene expression and reliable long-term bioprocess performance [25].

The aim of this study was to further improve ICA production by extending previous efforts that exploited mitochondrial and plasma-membrane transport systems for metabolic engineering, with the ultimate goal of developing a microbial strain suitable for industrial-scale bioprocessing. The metabolic engineering strategy primarily targeted the citrate membrane export protein, *Yl*Cex1, which belongs to the Major Facilitator Superfamily and functions as a drug:H^+^ antiporter in the plasma membrane. Inactivation of *YlCEX1* gene in *Y. lipolytica* completely abolished both CA and ICA production [9]. In this work, overexpression of *YlCEX1* gene in the ICA-producing strain YM4B253 generated strain YM4B2111 (W29 Δ*ku*70 Δ*Ylyhm2 YlSFC1 YlCEX1*), which exhibited a significant 1.7-fold increase in ICA titer compared with the parental strain (Fig. 1A; Table 1).

The consistent inversion of the intracellular CA/ICA ratio across all the strains containing the *YlYHM2* deletion indicates that disruption of the mitochondrial citrate carrier profoundly alters intracellular organic-acid homeostasis, driving the accumulation of ICA at the expense of CA. The phenotype was most pronounced in YM4B222, which accumulated the largest intracellular ICA pool (Fig. 1B). Considering that *YlSFC1* expression remains low during growth on glucose [8], this ICA pool is likely confined largely to the mitochondrial compartment. Importantly, overexpression of either *YlSFC1* or *YlCEX1* reduced intracellular ICA accumulation while simultaneously increasing ICA secretion (Fig. 1). These findings support the existence of an intracellular ICA reservoir, created by the *YlYHM2* deletion, that becomes accessible for secretion once transport capacity is enhanced. The greatest increase in ICA export was obtained when both transporters were co-expressed, highlighting the importance of coordinated mitochondrial and plasma-membrane transport in maximizing ICA efflux.

Although in the study by Erian et al. [9] overexpression of *YlCEX1* gene resulted in only a modest increase in secreted ICA relative to the wild‑type strain DSM 21175, the combined ICA + CA titres reported was extremely low. In contrast, the present work revealed a clear cooperative effect between *YlCEX1* and the mitochondrial transporter *YlSFC1* overexpression, resulting in substantially higher ICA production (Fig. 1A) and demonstrating the potential of this transport-engineering strategy for industrial bioprocesses. This improvement is likely attributable not only to enhanced transport capacity, which promotes product flux from the cytosol to the extracellular medium, but also to a reduction in intracellular ICA accumulation. By preventing the build-up of intracellular ICA pools, transporter overexpression may alleviate metabolic constraints on upstream pathway activity, although this possibility was not directly investigated in the present study. Collectively, these findings further emphasize the importance of coordinated transporter engineering as a powerful approach to maximize organic-acid secretion.

**Fig. 5:**
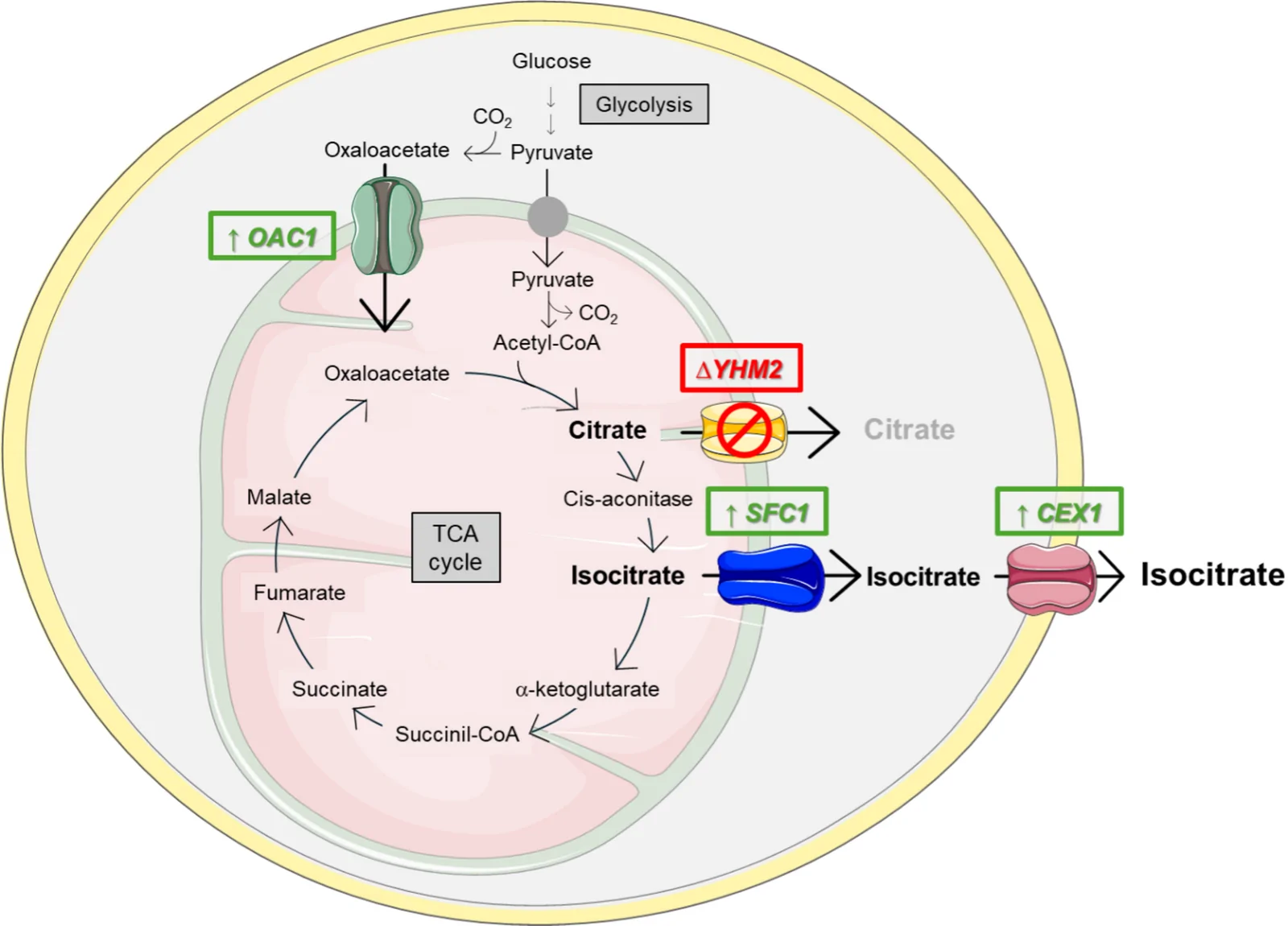
Schematic overview of the metabolic engineering strategy for ICA production in *Y. lipolytica* strain YM4B3122. The genetic modification included the inactivation of *YlYHM2* (indicated in red box), and the overexpression of *YlSFC1*, *YlCEX1* and *YlOAC1* genes (indicated in green boxes). *CEX1*, membrane citrate exporter; *OAC1*, oxaloacetate mitochondrial carrier; *SFC1*, succinate/fumarate carrier; *TCA* cycle, tricarboxylic acids cycle; *YHM2*, citrate-α-ketoglutarate mitochondrial carrier

Further improvement of the ICA‑producing strain focused on modulating the expression of mitochondrial carriers capable of replenishing the mitochondrial matrix with key intermediates of the TCA cycle. This approach provides an additional mechanism to reinforce precursor availability and sustain increased ICA biosynthesis in *Y. lipolytica*. Given that most mitochondrial organic‑acid transporters act as strictly coupled dicarboxylate and/or tricarboxylate exchangers and therefore cannot support a net increase in carbon entry into mitochondria, our efforts centered on *Yl*Oac1, *Yl*Dic1, and the *Yl*Mpc complex. These transporters represent the only known mitochondrial import systems in this yeast that can mediate net carbon uptake, as they supply OAA, dicarboxylates or pyruvate directly to the matrix (Fig. 2). Their ability to replenish TCA‑cycle precursors provides a rational means to elevate carbon availability at the early steps of the cycle, thereby supporting higher flux toward ICA formation. The genes encoding these transporters were introduced into the engineered background strain YM4B2111 (W29 Δ*ku*70 Δ*Ylyhm2 YlSFC1 YlCEX1*) either individually or in combination. This approach allowed us to evaluate how each individual carrier, as well as potential synergistic interactions among them, modulates intracellular metabolite routing and ultimately affects ICA secretion. Among all genetic modifications, overexpression of the oxaloacetate carrier gene *YlOAC1* (strain YM4B3122) (Fig. 5) was particularly effective, markedly increasing ICA production, although it also led to an undesired rise in CA levels (Fig. 3; Table 2).

In contrast, overexpression of the gene encoding the dicarboxylate carrier *Yl*Dic1 alone redirected carbon flux toward CA accumulation (Fig. 3). The underlying reason for this phenotype remains unclear, but it is likely associated with an imbalance in the pool of TCA-cycle intermediates. Notably, this imbalance was alleviated when *YlDIC1* gene and the *YlMPC1*/*YlMPC2* gene complex were co-expressed, supporting the idea that a coordinated supply of both acetyl-CoA and dicarboxylates is required to sustain efficient carbon flow from CA to ICA (Fig. 3). Another plausible explanation, though not directly tested experimentally, is that *Yl*Dic1 and *Yl*Sfc1 compete for cytosolic succinate. For *Yl*Sfc1, succinate is likely the most favorable counter-substrate for ICA transport, which would be consistent with the idea that competition for this metabolite could reduce transport efficiency of *Yl*Dic1, and contribute to the observed shift in carbon flux toward CA. This proposed mechanism, however, has not been directly validated and would require dedicated experiments, such as transporter kinetics or competition assays, to be confirmed.

In fungi, pyruvate carboxylase is located in the cytosol and therefore generates OAA and consequently malate outside the mitochondria. This metabolic organization implies that replenishment of the mitochondrial OAA pool relies primarily on transport processes rather than on intramitochondrial synthesis. De Jongh and Nielsen [26] demonstrated that increasing cytosolic malate levels, which can be transported into mitochondria to regenerate OAA, significantly enhances CA production in *Aspergillus niger*. Their findings highlighted the relevance of strengthening this anaplerotic node to boost flux through the OAA and acetyl‑CoA branch point. In our study, we reinforce the same metabolic node but through a different mechanism, promoting mitochondrial OAA import via the transporter *Yl*Oac1. *Yl* This approach may increase the intramitochondrial availability of OAA, thereby potentially enhancing flux through the OAA-acetyl CoA node and contributing to the observed increase in ICA biosynthesis in *Y. lipolytica*. However, this interpretation is based on the expected metabolic consequences of *OAC1* overexpression rather than on direct measurement of intramitochondrial OAA levels or flux through this node, and should therefore be regarded as a working hypothesis.

Unlike the strategy reported by Yuzbasheva et al. [8], the design of our ICA hyper‑producing strain did not include *YlAMPD* overexpression, since this modification did not increase the ICA titer in our strain background (Figure S2, Supplementary Materials). *AMPD* converts adenosine monophosphate (AMP) into inosine monophosphate (IMP), thereby lowering the intracellular AMP pool. Because fungal *IDH* is allosterically activated by AMP, reducing AMP levels can diminish *IDH* activity and consequently limit the diversion of ICA toward the TCA cycle, in principle favoring ICA accumulation. Previous studies on CA production support this interpretation. In Yuzbasheva et al. [7], *YlAMPD* overexpression alone yielded only a minor increase in citrate production from glucose. A substantial enhancement was observed only when *YlAMPD* gene was co‑overexpressed with the mitochondrial carrier *YlYHM2* gene, which nearly doubled CA production relative to the wild‑type strain. These findings suggest that *YlAMPD* can positively influence organic acids production only when mitochondrial transport capacity is concurrently strengthened.

Overall, the available evidence suggests that achieving high ICA titres in *Y. lipolytica* depends primarily on the modulation of transport systems rather than on altering AMP metabolism. Both mitochondrial carriers and membrane associated transporters appear to exert greater control over ICA flux than the activities of biosynthetic or regulatory enzymes. This aligns with the metabolic architecture of *Y. lipolytica*, a yeast naturally predisposed to overproduce CA and ICA, where targeted tuning of transporter expression efficiently channels carbon through the TCA cycle and elevates ICA output.

Bench-scale cultivation of the ICA-overproducing strain YM4B3122 (W29 Δ*ku*70 Δ*Ylyhm2 YlSFC1 YlCEX1 YlOAC1*) (Fig. 5), which displayed in flask cultivations the highest ICA titer and molar yield among all the recombinant strains (Table 2), was carried out in a bioreactor alongside the WT strain, using a glucose pulse-feeding strategy (Fig. 4). The fed-batch cultivation strategy with a pulse-feeding approach adopted in this study was highly effective in supporting high-level ICA production. Strain YM4B3122 reached an ICA titer of 152.76 g/L, with a production selectivity of 95% corresponding to an ICA/CA ratio of 20. These values represent a substantial improvement compared with our previously engineered strain which achieved a maximum ICA titer of 136.7 g/L with 88% selectivity within the secreted ICA-CA mixture in bioreactor cultivation [8]. The higher titer and selectivity achieved in the present work highlight the effectiveness of the engineered YM4B3122 strain combined with the optimized fed-batch pulse-feeding strategy.

Further improvements of the bioprocess could be achieved through the implementation of more controlled feeding strategies, such as linear or exponential glucose feeding profiles. These approaches could provide tighter control over substrate availability and improve process robustness during large-scale cultivation while maintaining the nitrogen-limited conditions required for ICA production. In addition, the integration of feedback-based strategies, such as dissolved oxygen (DO)-stat control, could provide an effective tool to dynamically adjust carbon feeding according to cellular metabolic activity, thereby improving process robustness and scalability. In this perspective, the high ICA titer and selectivity achieved by strain YM4B3122 highlight the strong potential of this engineered platform for the development of industrial ICA production processes.

## Conclusions

In conclusion, the results presented in this study highlight how precise engineering of mitochondrial and plasma membrane transport systems without modification of enzymatic steps can effectively redirect TCA-derived carbon flux toward ICA biosynthesis in *Y. lipolytica*. The integration of targeted genetic modifications with optimized bioprocess conditions enabled the development of a strain capable of reaching the highest ICA titer and selectivity reported to date.

## Materials and methods

### Strains and culture media

*Escherichia coli* DH5α used for plasmid construction, propagation and amplification, was cultivated in Luria Bertani (LB) media at 37 °C with shaking (200 rpm). This medium was supplemented with antibiotics according to the resistance carried by the plasmids, either 50 µg/mL spectinomycin (Sigma-Aldrich, St. Louis, MO, USA) or 100 µg/mL ampicillin (Sigma-Aldrich, St. Louis, MO, USA), to allow selective growth. All *Y. lipolytica* strains constructed in this study are summarised in Table 3 and all of them are derived from Y-4973, an uracil auxotroph and *Ku70*-mutant (Δ*ura3* Δ*ku70*) obtained from the W29 strain (MatA, wild-type), that shows an increased rate of homologous recombination [11]. *Y. lipolytica* strains were cultivated in different liquid media at 28 °C and with orbital shaking at 200 rpm. The rich medium, used for seed culture, was YPD medium composed of 10 g/L yeast extract (Biokar Diagnostics, Allonne, France), 20 g/L peptone (Biokar Diagnostics, Allonne, France), and 20 g/L glucose (Sigma-Aldrich, St. Louis, MO, USA). Whenever required, the medium was supplemented with 350 µg/mL nourseothricin (Jena Bioscience, Jena, Germany) for the overnight growth of *Y. lipolytica* strains transformed with Cas9 expressing plasmids, or with 450 µg/mL hygromycin (Sigma-Aldrich, St. Louis, MO, USA) for selective marker recovery procedure. The minimal medium was composed of YNB (1.7 g/L Yeast Nitrogen Base without nitrogen source, Difco™, Becton Dickinson, Sparks, MD, USA) supplemented with glucose and (NH_4_)_2_SO_4_ (VWR Chemicals, Radnor, PA, USA) at various concentrations based on the scope of the cultivation. For uracil auxotrophic strains, the minimal medium was supplemented with 0.3 g/L uracil (Sigma-Aldrich, St. Louis, MO, USA). For selection of *URA3* marker-based transformants, the minimal medium consisted of 1.7 g/L YNB, 5 g/L (NH_4_)_2_SO_4_, 20 g/L glucose, and 2 g/L of each of the 20 proteinogenic amino acids (Sigma-Aldrich, St. Louis, MO, USA), without uracil. Solid media were prepared by adding 20 g/L of bacteriological agar (Type E, Biokar Diagnostics, Allonne, France).

**Table 3:** *Y. lipolytica* strains used in this study and their genotype

Strain	Genotype	Reference
Y-4973 (WT)	W29 (Δ*ku*70 Δ*ura3*)	[11]
YM4B222	Y-4973 ∆*Ylyhm2* Δ*ura3*	[28]
YM4B253	YM4B222 IntF9: URA3-pTEF-*YlSFC1*-tLIP2	This study
YM4B277	YM4B253 Δ*ura3*	This study
YM4B662	YM4B222 IntC2: URA3-pTEF-*YlCEX1*-t*Sc*PGK1	This study
YM4B2111	YM4B277 IntC2: URA3-pTEF-*YlCEX1*-t*Sc*PGK1	This study
YM4B3111	YM4B2111 Δ*ura3*	This study
YM4B3122	YM4B3111 IntB11: URA3-pEXP1-*YlOAC1*-t*Sc*ADH1	This study
YM4B5123	YM4B3111 IntD6: URA3-pTEF-*YlDIC1*-tLIP2	This study
YM4B5717	YM4B3111 IntE15: URA3-pGAPDH-*YlMPC1*-t*Sc*PGK1/ pTEF-*YlMPC2*-t*Sc*ENO2	This study
YM4B424	YM4B3122 Δ*ura3*	This study
YM4B443	YM4B424 IntE15: URA3-pGAPDH-*YlMPC1*-t*Sc*PGK1/ pTEF-*YlMPC2*-t*Sc*ENO2	This study
YM4B5719	YM4B3111 IntC14: URA3-pGAPDH-*YlMPC1*-t*Sc*PGK1/ pTEF-*YlMPC2*-t*Sc*ENO2/pTEF1-*YlDIC1*-tLIP2	This study
YM4B455	YM4B443 Δ*ura3*	This study
YM4B464	YM4B455 IntF11: URA3-pTEF-*YlSFC1*-tLIP2	This study
YM4B481	YM4B424 IntE8: URA3-pTEF-*YlAMPD*-t*Sc*PGK1	This study

Genotypes are presented in the format [integration site]: [promoter]-[gene]-[terminator], where the integration site (Int) refers to the specific genomic locus targeted, as described by Yuzbashev et al. [11]. The promoter (p) and terminator (t) elements vary depending on the expression cassette used. The overexpressed gene is shown in italics

### Construction of plasmids and recombinant strains

Plasmids for yeast genome engineering were constructed using vectors from the synthetic biology toolkit *YaliCraft®* [11]. For the overexpression of genes *YlSFC1* (YALI0E34672g), *YlCEX1* (YALI0D20196g), *YlOAC1* (YALI0E04048g), *YlDIC1* (YALI0B03344g), *YlMPC1*/*YlMPC2* (YALI0F00264g and YALI0C03223g) and *YlAMPD* (YALI0E11495g) plasmids were constructed using the Golden Gate assembly method [27], as previously described by Ciliberti et al. [28]. The overexpression cassettes were released from the plasmids by digestion with the *NotI* endonuclease; the corresponding donor DNA was co-transformed with the Cas9-helper plasmid, targeting a specific locus in the *Y. lipolytica* genome. The *URA3* gene served as the selection marker for the overexpression constructs, making the transformed strains prototrophic for uracil. For each subsequent step, an auxotrophic derivative was obtained using the Cre-lox 66/71 recombination system, as previously described [11]. To verify the correct chromosomal integration, PCR was performed using primers annealing to the flanking regions of the integration locus (Table S2, Supplementary Materials). The complete set of plasmids used in this study is provided in Table S3 (Supplementary Materials).

### Shake-flask cultures

Seed culture from YPD plates was grown overnight in 10 mL of YPD medium at 28 °C and 200 rpm. Pre-cultures were centrifuged, washed with sterile distilled water, and used for inoculation at initial optical density (OD_600nm_) equal to 0.1 into 250-mL baffled flasks containing 50 mL minimal medium composed of 1.7 g/L YNB supplemented with 90 g/L glucose and 2 g/L (NH_4_)_2_SO_4_ (C/N 85). The medium pH was buffered by adding sterile 10 g/L CaCO_3_ after 5–6 h from the inoculum. Cultivations were performed for up to 4 days at 28 °C on an orbital shaker operating at 200 rpm. Samples were collected at regular time intervals for OD_600_, pH and HPLC analysis. All measurements were performed using at least two biological replicates, and average values with standard errors were calculated.

### Bioreactor fermentations

Fed-batch fermentation was conducted in a 5 L bioreactor (BIOSTAT B Plus; Sartorius, Germany) with a starting working volume of 1 L. Bioreactor medium was composed of 30 g/L glucose; 3 g/L (NH_4_)_2_SO_4_; 1.7 g/L YNB; dissolved in tap water. The seed culture was grown for 24 h at 28 °C and 200 rpm, in a 250-mL baffled flask containing 50 mL of YPD medium. The bioreactor was inoculated at an initial OD_600_ 1.0. The bioreactor process was conducted for 213 h at 28 °C, with agitation rate of 800 rpm, aeration rate of 0.8 vvm (0.8 L/min) and pH 6.0 maintained by the automatic addition of a 400 g/L KOH solution. During the cultivation, the bioreactor was fed approximately every 24 h with sterile solutions of 600 g/L glucose and 200 g/L (NH_4_)_2_SO_4_. The glucose solution was added based on the estimated consumption in the previous 24 h with an increase of 10%; the (NH_4_)_2_SO_4_ solution was added in order to maintain a C/N ratio of 94. For the auxotrophic wild-type (WT) strain, Y-4973 (W29 Δ*ku70* Δ*ura3*), the medium was also supplemented with 0.3 g/L uracil at the beginning and then every 48 h of cultivation in order to allow the growth of the strain. A silicone-based antifoaming agent was added to the cultures at a final concentration of 0.1% (*v/v*). Prior to inoculation, all media were sterilized at 121 °C for 30 min.

### Analytical methods

HPLC analysis was performed to quantify the amount of organic acids and glucose. The flask cultivations were monitored by collecting the supernatant, after adding 1 M HCl solution to neutralize CaCO_3_. The bioreactor cultivation was monitored by sampling 10 mL every 24 h. Prior to analysis, culture supernatants were centrifuged at 12.000 rpm for 10 min and diluted when necessary. The HPLC system used was a Nexera Lita (Shimadzu, Japan), equipped with a photodiode array detector (Photodiode Array Mod. Shimadzu SPD-M40), and a refractive index detector (Shimadzu RID- 20 A). For organic acids detection, a Kinetex EVO C18 column (150 mm x 4.6 mm, 100 Å, 5 μm) (Phenomenex Inc., USA) was used with 0.1% v/v H_3_PO_4_ at pH 1.5 as the mobile phase, coupled to a PDA detector set at 210 nm. For glucose measurement, a Rezex ROA-Organic Acid H+ (8%) column (300 mm x 7.8 mm) (Phenomenex Inc., USA) was used with 0.0025 M H_2_SO_4_ as the mobile phase and a refractive index detector. Peaks were identified by comparison with reference standards dissolved in ultrapure water. Calibration curves for peak quantification were constructed over a concentration range of 0.1–30 g/L.

To determine dry cell weight (DCW), cultures were washed, frozen, and lyophilized in a pre-weighted tube using a Labconco FreeZone 2.5 L Benchtop Freeze Dryer operating at -50 °C and 0.05 mbar for 48 h. The weight differences corresponded to the mg of cells present in the dried culture volume.

### LC-MS analysis of intracellular isocitrate and citrate

Metabolites were extracted from 2 mg of lyophilized biomass by resuspension in 500 µL of methanol/water (1:1, v/v). Cells were disrupted by three freeze–thaw cycles to promote mechanical lysis, then pelleted by centrifugation at 15.000 rpm for 10 min at 4 °C [29]. A 100 µL aliquot of the supernatant was transferred to a separate microcentrifuge tube and evaporated to dryness in a vacuum concentrator. The dried extract was reconstituted in 75 µL of methanol/water (3:17, v/v), briefly vortexed and centrifuged at 11.000 rpm for 10 min at 4 °C.

LC–MS analysis was performed on a Vanquish UHPLC system coupled to an Orbitrap Exploris 240 mass spectrometer (Thermo Fisher Scientific). Chromatographic separation was carried out on a Kinetex C18 column (150 mm length × 2.1 mm internal diameter, 2.6 μm particle size; Phenomenex) maintained at 30 °C. Mobile phase A was LC–MS grade water with 0.2% formic acid, and mobile phase B was 100% methanol. The elution gradient was as follows: 0–4 min, 3.5% B; 4–14 min, linear increase to 80% B; 14–15 min, linear increase to 98% B; 15–21 min, held at 98% B; 21–21.5 min, returned to 3.5% B; 21.5–27 min, held at 3.5% B for re-equilibration [30]. The flow rate was 150 µL/min, and the injection volume was 1 µL.

Mass spectrometric data were acquired in separate positive- and negative-ion heated electrospray ionization (H-ESI) modes with the following source parameters: spray voltage, 3.4 kV (positive) and 3.0 kV (negative); ion transfer tube temperature, 280 °C; vaporizer temperature, 100 °C; sheath gas flow, 35 arbitrary units (a.u.); auxiliary gas, 10 a.u.; sweep gas, 0 a.u. Full-scan spectra were acquired on the Orbitrap analyzer over m/z 50–750 at a resolving power of 120.000, with the RF lens set to 70%. Instrument control and data acquisition were performed with Xcalibur software (Thermo Fisher Scientific). Citric acid and isocitric acid were acquired in negative-ion mode and quantified by external calibration. 

### Bioinformatic analysis

The phylogenetic tree was built using 9 protein sequences of *Y. lipolytica* and 11 of *Saccharomyces cerevisiae*, corresponding to di- and tri-carboxylate mitochondrial transporters and MPC complex. The phylogenetic tree was designed using PhyML v3.1 in SeaView4 [31] from a Muscle multiple-sequence alignment and drawn in iTOL v.6 [32]. The genes coding for putative pyruvate transporter (MPC complex) of *Y. lipolytica* were identified by on-line BLASTp search [33] using the sequences of *S. cerevisiae Sc*Mpc1, *Sc*Mpc2 and *Sc*Mpc3 as queries. Amino acid sequences were aligned by on-line tool NPS@: Network Protein Sequence Analysis [34]. The identity matrix was created by using the web tool SIAS Sequence Identity And Similarity [35].

### Data analysis

All experiments were performed in at least two biological replicates and results are presented as average ± standard error to ensure statistical reliability. Graphical data visualization and statistical analyses were performed using GraphPad Prism v.10. Differences between experimental groups were assessed using the unpaired Student’s *t*-test, with significance defined as P-value < 0.05 (*P < 0.05; **P < 0.01; ***P < 0.001).

## Supplementary Information

Below is the link to the electronic supplementary material.

Supplementary Material 1

## Data Availability

All data generated or analyzed during this study are included in this published article and its supplementary information files. The engineered strain YM4B3122 (DSM35454) is available from the DSMZ culture collection under accession number DSM35454. Additional materials or datasets are available from the corresponding author upon reasonable request.

## Abbreviations

AMPD: Adenosine monophosphate deaminase
AMP: Adenosine monophosphate
CA: Citric acid
C/N: Carbon-to-nitrogen ratio
CEX1: Plasma membrane citrate/isocitrate exporter
DCW: Dry cell weight
DIC1: Mitochondrial dicarboxylate carrier
ICA: Isocitric acid
ICA/CA: Isocitrate-to-Citrate ratio
IDH: Isocitrate Dehydrogenase
IMP: Inosine monophosphate
LB: Luria Bertani
MPC: Mitochondrial pyruvate carrier
NHEJ: Non‑Homologous End Joining
OAA: Oxaloacetate
OAC1: Mitochondrial oxaloacetate carrier
SFC1: Succinate/Fumarate carrier
Y_P/S_: Yield of product on substrate
YHM2: Mitochondrial citrate–α ketoglutarate carrier
YNB: Yeast nitrogen base
TCA: Tricarboxylic Acid cycle
URA3: Orotidine-5’-phosphate decarboxylase marker
WT: Wild-type
