# Correction: Mitochondrial and plasma‑membrane transport‑based engineering enables industrial‑level isocitric acid production in *Yarrowia lipolytica*

**DOI:** 10.1186/s12934-026-03156-x

**Published:** 2026-11-02

**Authors:** Eugenia Messina, Cosetta Ciliberti, Evgeniya Yuzbasheva, Serena Barile, Tigran Yuzbashev, Zbigniew Lazar, Ivan Laptev, Ferdinando Palmieri, Luigi Palmieri, Isabella Pisano, Gennaro Agrimi

**Affiliations:** 1 Department of Biosciences, Biotechnology and Environment, University of Bari Aldo Moro, Campus Universitario, via Orabona 4, Bari, 70125 Italy; 2 Both Strands Ltd, St Albans, Hertfordshire, AL1 4QW UK; 3 Rothamsted Research, Harpenden, AL5 2JQ UK; 4 Department of Biotechnology and Food Microbiology, Faculty of Biotechnology and Food Science, Wrocław University of Environmental and Life Sciences, Chelmonskiego 37, Wrocław, 51-630 Poland; 5 Alterhof LLC, Melnikova 3, Moscow, 109316 Russian Federation; 6 Interuniversity Consortium for Biotechnology (CIB), Trieste, 34100 Italy

**Correction: Microbial Cell Factories (2026) 25:187**

**https://doi.org/10.1186/s12934-026-03098-4**

In this article, Figures 3 and 4 appeared overlapped. This has now been corrected in the original article.

In this article, Table 3, which is referenced in the Materials and Methods section, was incorrectly placed on page 7 instead of page 12. This has now been corrected in the original article.

The original article has been corrected.

