# Supplementary material for: Mitochondrial and plasma‑membrane transport‑based engineering enables industrial‑level isocitric acid production in Yarrowia lipolytica
Source: Microb Cell Fact. 2026 Aug 26;25:187. doi: 10.1186/s12934-026-03098-4 (PMC13602682; doi:10.1186/s12934-026-03098-4)
Supplement: Supplementary file 1 [file 12934_2026_3098_MOESM1_ESM.docx]

**Mitochondrial and plasma‑membrane transport‑based engineering enables industrial‑level isocitric acid production in *Yarrowia lipolytica***

Eugenia Messina^*^, Cosetta Ciliberti^*^, Evgeniya Yuzbasheva, Serena Barile, Tigran Yuzbashev, Zbigniew Lazar, Ivan Laptev, Ferdinando Palmieri, Luigi Palmieri, Isabella Pisano, Gennaro Agrimi

* These authors contributed equally to this work

**Supplementary Materials**

**Additional Tables**

**Table S1.** Percentage of amino acidic identity among Mpc subunits of *Saccharomyces cerevisiae* (*Sc*Mpc1, *Sc*Mpc2 and *Sc*Mpc3) and the two putative Mpc subunits of *Yarrowia lipolytica* (YALI0F00264p and YALI0C03223p). The identity matrix was created using the web tool “SIAS Sequence Identity And Similarity”.

| *Percentage of identity (%)* | ***Sc*Mpc2** | ***Sc*Mpc3** | **YALI0C03223p** | ***Sc*Mpc1** | **YALI0F00264p** |
| --- | --- | --- | --- | --- | --- |
| ***Sc*Mpc2** | 100 | 76.74 | 56.58 | 34.1 | 33.06 |
| ***Sc*Mpc3** | 76.74 | 100 | 58.95 | 33.84 | 36.29 |
| **YALI0C03223p** | 56.58 | 58.95 | 100 | 36.92 | 36.29 |
| ***Sc*Mpc1** | 34.1 | 33.84 | 36.92 | 100 | 63.7 |
| **YALI0F00264p** | 33.06 | 36.29 | 36.29 | 63.7 | 100 |

**Table S2.** Primers used and their sequences.

| **Primer name** | **Sequence (5’->3’)** | **Description** |
| --- | --- | --- |
| IntF9-chr-F | GTCACGTAAACATTCCAGCACGC | Verification of *YlSFC1* integration in IntF9 |
| IntF9-chr-R | TGGAGGATGACTACGGTTACAGATAGTGG | Verification of *YlSFC1* integration in IntF9 |
| IntC2-chr-F | GTGTGTCCAACCAAAGTGAG | Verification of *YlCEX1* integration in IntC2 |
| IntC2-chr-R | GAGAGACATTGTTGTGATTGAC | Verification of *YlCEX1* integration in IntC2 |
| IntB11-chr-F | GCAGTTTAGCATCCCTCAAG | Verification of *YlOAC1* integration in IntB11 |
| IntB11-chr-R | CTCTTCTCTTATCATGCAGCAG | Verification of *YlOAC1* integration in IntB11 |
| IntD6-chr-F | AGGTGGTCAATGAGTTGTCG | Verification of *YlDIC1* integration in IntD6 |
| IntD6-chr-R | TACGTGTGTTTGGCTGTCTC | Verification of *YlDIC1* integration in IntD6 |
| IntE15-chr-F | ACCTACCTCCTTCTATCTCC | Verification of *YlMPC1-MPC2* integration in IntE15 |
| IntE15-chr-R | TCTGTAAGCTGAAGCACATTC | Verification of *YlMPC1-MPC2* integration in IntE15 |
| IntC14-chr-F | GTTCTGTAGTCGCCAACATG | Verification of  *YlMPC1-MPC2-YlDIC1* integration in IntC14 |
| IntC14-chr-R | ATGGAGTAGGTAACGTGACCG | Verification of  *YlMPC1-MPC2-YlDIC1* integration in IntC14 |
| IntE8-chr-F | GAAGCCACCAAAGACACGATCCAGC | Verification of *YlAMPD* integration in IntE8 |
| IntE8-chr-R | GGAGAGCGATGAAGTCTTTAGGCACTG | Verification of *YlAMPD* integration in IntE8 |
| IntF11-chr-F | CGCATAGTTTCTGTCTCCCC | Verification of *YlSFC1* integration in IntF11 |
| IntF11-chr-R | TCAAGTTCTTGCACTCTGTTG | Verification of *YlSFC1* integration in IntF11 |

**Table S3.** List of plasmids used in this study.

| **Plasmid ID** | **Description** | **Purpose** | **Reference** |
| --- | --- | --- | --- |
| p-M4B231 | pF9US1.3-pTEF-*YlSFC1-*tLIP2 (*URA3*) | Overexpression of *YlSFC1* gene in F9 integration site | This study |
| p-1104 | pCasNA-IntF9 | Expression of Cas9 and gRNA targeting F9 integration site | Yuzbashev et al., 2023 |
| p-621 | pCreHA | Recovery of marker URA3 | Yuzbashev et al., 2023 |
| p-2090 | pC2US1.1-pTEF-*YlCEX1*-t*Sc*PGK1 (*URA3*) | Overexpression of *YlCEX1* gene in C2 integration site | This study |
| p-1426 | pCasNA-IntC2 | Expression of Cas9 and gRNA targeting C2 integration site | Yuzbashev et al., 2023 |
| p-M4B3101 | pB11US1.1-pEXP1-*YlOAC1*-t*Sc*ENO2 (*URA3*) | Overexpression of *YlOAC1* gene in B11 integration site | This study |
| p-1249 | pCasNA-IntB11 | Expression of Cas9 and gRNA targeting B11 integration site | Yuzbashev et al., 2023 |
| p-M4B5114 | pD6US1.1-pTEF-*YlDIC1*-tLIP2 URA3 | Overexpression of *YlDIC1* gene in D6 integration site | This study |
| p-1133 | pCasNA-IntD6 | Expression of Cas9 and gRNA targeting D6 integration site | Yuzbashev et al., 2023 |
| p-M4B434 | pE15UA2.2-pGAPDH-*YlMPC1*-t*Sc*PGK1/pTEF-*YlMPC2*-t*Sc*ENO2 (*URA3*) | Overexpression of *YlMPC1* and *YlMPC2* genes in E15 integration site | This study |
| p-1136 | pCasNA-IntE15 | Expression of Cas9 and gRNA targeting E15 integration site | Yuzbashev et al., 2023 |
| p-M4B563 | pC14UA2.3- pGAPDH-*YlMPC1*-t*Sc*PGK1/pTEF-*YlMPC2*-t*Sc*ENO2/pTEF1-*YlDIC1-*tLIP2 (*URA3*) | Overexpression of *YlMPC1*, *YlMPC2* and *YlDIC1* genes in C14 integration site | This study |
| p-1100 | pCasNA-IntC14 | Expression of Cas9 and gRNA targeting C14 integration site | Yuzbashev et al., 2023 |
| p-M4B431 | pF11US1.1-pEXP1-*YlSFC1*-tLIP2 (*URA3*) | Overexpression of *YlSFC1* gene in F11 integration site | This study |
| p-1135 | pCasNA-IntF11 | Expression of Cas9 and gRNA targeting F11 integration site | Yuzbashev et al., 2023 |
| p-M4B421 | pE8US1.1-pTEF-Yl*AMPD*-t*Sc*PGK1 (*URA3*) | Overexpression of *YlAMPD* gene in E8 integration site | This study |
| p-910 | pCasNA-IntE8 | Expression of Cas9 and gRNA targeting E8 integration site | Yuzbashev et al., 2023 |

**Additional Figures**

**Figure S1.** Isocitric and citric acid production, after 4 days of cultivation in 250-mL baffled flasks with 50 mL YNB+Glucose 90g/L and nitrogen starvation [(NH_4_)_2_SO_4_ 2 g/L], of the strains YM4B443 (blue) and YM4B464 (pink). Data represent the mean of two independent experiments, with error bars indicating the standard error.

**Figure S2.** Isocitric and citric acid production, after 4 days of cultivation in 250-mL baffled flasks with 50 mL YNB+Glucose 90g/L and nitrogen starvation [(NH_4_)_2_SO_4_ 2 g/L], of the strains YM4B3122 (purple) and YM4B481 (brown). Data represent the mean of at least three independent experiments, with error bars indicating the standard error.

**Figure S3**. Fed-batch bioreactors cultivations of YM4B3122 ICA-overproducing strain (**A**) and of WT strain Y-4973 (**B**). These cultivations were carried at 28 °C with agitation rate of 800 rpm, air flow of 0.8 vvm and pH set at 6, inoculated with an initial OD_600nm_ of 1.0. Graph of the second replicate.
